# Biomarkers of Environmental Enteropathy, Inflammation, Stunting, and Impaired Growth in Children in Northeast Brazil

**DOI:** 10.1371/journal.pone.0158772

**Published:** 2016-09-30

**Authors:** Richard L. Guerrant, Alvaro M. Leite, Relana Pinkerton, Pedro H. Q. S. Medeiros, Paloma A. Cavalcante, Mark DeBoer, Margaret Kosek, Christopher Duggan, Andrew Gewirtz, Jonathan C. Kagan, Anna E. Gauthier, Jonathan Swann, Jordi Mayneris-Perxachs, David T. Bolick, Elizabeth A. Maier, Marjorie M. Guedes, Sean R. Moore, William A. Petri, Alexandre Havt, Ila F. Lima, Mara de Moura Gondim Prata, Josyf C. Michaleckyj, Rebecca J. Scharf, Craig Sturgeon, Alessio Fasano, Aldo A. M. Lima

**Affiliations:** 1 University of Virginia School of Medicine (Division of Infectious Diseases and International Health, Department of Medicine, Department of Pediatrics and Center for Global Health), Charlottesville, VA, United States of America; 2 Clinical Research Unit, Federal University of Ceara, Fortaleza, Brazil; 3 Division of Gastroenterology at Boston Children’s Hospital, Harvard University, Boston, MA, United States of America; 4 Institute for Biomedical Sciences in the Center for Inflammation, Immunity and Infection at Georgia State University, Atlanta, GA, United States of America; 5 Mucosal Immunology and Biology Research Center and Division of Pediatric Gastroenterology and Nutrition at Massachusetts General Hospital for Children, Harvard University, Boston, MA, United States of America; 6 Imperial College, London, United Kingdom; 7 Cincinnati Children’s Hospital, Cincinnati, OH, United States of America; 8 Johns Hopkins Bloomberg School of Public Health, Baltimore, MD, United States of America; University of Chicago Medical Center, UNITED STATES

## Abstract

Critical to the design and assessment of interventions for enteropathy and its developmental consequences in children living in impoverished conditions are non-invasive biomarkers that can detect intestinal damage and predict its effects on growth and development. We therefore assessed fecal, urinary and systemic biomarkers of enteropathy and growth predictors in 375 6–26 month-old children with varying degrees of malnutrition (stunting or wasting) in Northeast Brazil. 301 of these children returned for followup anthropometry after 2-6m. Biomarkers that correlated with stunting included plasma IgA anti-LPS and anti-FliC, zonulin (if >12m old), and intestinal FABP (I-FABP, suggesting prior barrier disruption); and with citrulline, tryptophan and with lower serum amyloid A (SAA) (suggesting impaired defenses). In contrast, subsequent growth was predicted in those with higher fecal MPO or A1AT and also by higher L/M, plasma LPS, I-FABP and SAA (showing intestinal barrier disruption and inflammation). Better growth was predicted in girls with higher plasma citrulline and in boys with higher plasma tryptophan. Interactions were also seen with fecal MPO and neopterin in predicting subsequent growth impairment.

Biomarkers clustered into markers of 1) functional intestinal barrier disruption and translocation, 2) structural intestinal barrier disruption and inflammation and 3) systemic inflammation. Principle components pathway analyses also showed that L/M with %L, I-FABP and MPO associate with impaired growth, while also (like MPO) associating with a systemic inflammation cluster of kynurenine, LBP, sCD14, SAA and K/T. Systemic evidence of LPS translocation associated with stunting, while markers of barrier disruption or repair (A1AT and Reg1 with low zonulin) associated with fecal MPO and neopterin.

We conclude that key noninvasive biomarkers of intestinal barrier disruption, LPS translocation and of intestinal and systemic inflammation can help elucidate how we recognize, understand, and assess effective interventions for enteropathy and its growth and developmental consequences in children in impoverished settings.

## Introduction

Children living in impoverished areas around the world often develop stunted growth from 4 to 24 months of age, a concern that is heightened by potential lasting consequences of impaired cognitive development [1–5]. Increasingly sensitive detection methods are revealing that multiple and repeated intestinal infections are even more common (with and without overt diarrheal illnesses) in young children living in impoverished areas lacking in adequate sanitation or clean water [6, 7]. These heavy pathogen burdens may associate with growth faltering or cognitive impairment [8–14]. Undernutrition is also common and may further worsen the impact of enteric infections on growth and development, leading to a potential “vicious cycle” of malnutrition and enteric infections [15–17]. These potential relationships can be causally dissected in animal models, where certain enteric infections, such as *Cryptosporidium* or enteroaggregative *Escherichia coli* [18, 19] or even certain mixed Bacteroidetes and Proteobacteria [20] can *further* worsen the growth impairment and intestinal damage in undernourished states. In the latter case, a malnourishing diet resulted in growth failure and microbiome alterations, but without intestinal damage unless certain mixed microbiota were added. This problem of environmental enteropathogens inducing enteric dysfunction has been recognized for over half a century in tropical, developing areas and among Peace Corps volunteers [15, 21, 22]. More recently this clinical entity has been associated with the concept of disruption of gut barrier function, passage of microorganisms and/or their bioproducts from the intestinal lumen to the lamina propria, and mucosal inflammation, ultimately leading to damage of villous architecture and further impaired digestive and absorptive functions and barrier disruption, thereby generating a vicious cycle of impaired gut functions referred to as “Environmental Enteropathy” (EE) [23]. Numerous extensive studies have suggested that different assessments of intestinal barrier disruption, local and systemic inflammatory responses can associate with not only impaired growth, but also with poor household environments that likely predispose to enteric infections and EE [24–29]. Yet this hugely important problem for healthy child growth and development remains poorly understood [30]. Our objective therefore was to examine a wide range of potential fecal, urinary and plasma biomarkers to determine how they associate with each other and with malnutrition (defined by height or weight for age) and to determine which ones best predict impaired subsequent growth.

The need is great for biomarkers that can predict both EE onset and its longer term growth and developmental impact as well as to evaluate potential interventions designed to ameliorate these mechanisms and improve clinical outcome. We therefore examined potential biomarkers that might associate functional and structural “enteropathy” with malnutrition or with subsequent growth impairment in children enrolled in a study of malnutrition at a nutrition clinic serving several impoverished communities in and near Fortaleza, Ceará in Northeast Brazil. In particular, we assessed potential plasma, urine and fecal biomarkers of intestinal epithelial or barrier disruption, evidence of bacterial product translocation and intestinal and systemic inflammatory responses, and intestinal permeability.

## Methods

### Study location and population

The study was conducted at Promotion of Nutrition and Human Development (IPREDE) clinic located in Fortaleza, CE, Brazil. Details of the geographic location, population, demographics, environmental and socioeconomic status have been described elsewhere [31]; this outpatient clinic serves children from several impoverished communities in and near Fortaleza, often with moderate or severe malnutrition, having weight-for-age z-scores and height-for-age z-scores (WAZ or HAZ) ranging from -2 to -6. Standard nutrition education and supplementation was provided according to local standards based on World Health Organization guidelines [32]. The study started in Aug, 2010 and ended with follow up in Sep, 2013. Malnourished or “case” children were initially defined as having WAZ scores <-2 and, to the extent possible, age and gender matched ‘non-malnourished controls’ were defined as having a WAZ better than -1. However, others have correlated linear growth (or HAZ) with EE or other outcomes such as cognitive impairment [3]; hence we have examined both WAZ and HAZ in these analyses. Children were excluded if they had underlying recognized disease (other than malnutrition) or if they did not have a responsible parent or guardian ≥16 years old. After obtaining informed consent from the responsible parent or guardian, a total of 402 children aged 6–26 months (201 malnourished and 201 age and gender-matched non-malnourished) were enrolled between August 30, 2010 and July 12, 2013; 375 provided fecal and blood samples and completed a lactulose-mannitol absorption test, and were asked to return for followup in 2–6 months.

The MAL-ED case-control study protocol and consent forms were approved by the local institutional review board (IRB) at Universidade Federal do Ceará, the national IRB, Conselho Nacional de Ética em Pesquisa, Brasília, DF, Brazil, and the IRB at the University of Virginia, VA. The consent forms were reviewed and signed by the responsible parents or caregivers at the time of the screening process of the study protocol.

### Anthropometry and biomarker assessments and statistical analyses

Detailed methods for anthropometric measurements, assessment of blood, stool, and urine biomarkers and statistical methods are provided in S1 File. As shown in Table 1, 375 children were enrolled and had initial anthropometry and were invited to provide a fecal specimen, have a L/M absorption test and to have blood obtained for testing potential biomarkers of intestinal barrier function, intestinal and systemic inflammation and injury repair. Shown in Table 2 are the 13 plasma, 4 fecal and urinary tests with at least 274 valid results obtained from within 1 month of enrollment. Markers of barrier function included urinary L/M absorption, fecal A1AT, Reg-1, and plasma LPS (acute levels, by neutralization luminescence [LUM] assay), IgG and IgA anti-LPS anti-FliC, zonulin, I-FABP and, with limited amounts of plasma available, claudin-15. Fecal MPO and neopterin were tested to assess intestinal inflammation, and plasma SAA, sCD14, LBP, citrulline, tryptophan and kynurenine were measured to assess systemic inflammatory responses and as potential predictors of intestinal injury repair. These 18 markers of intestinal barrier disruption and inflammation had adequate samples for assay in at least 274 (to 321) children at enrollment for study of associations with enrollment stunting (or wasting). Of several fecal biomarkers that have been used to assess intestinal inflammation, we selected myeloperoxidase (MPO) as our main test fecal marker of acute neutrophilic intestinal inflammation because of its ready availability, relatively less influence by age or breast-feeding and its potential use in murine models of enteropathy. In a separate analysis comparing fecal MPO, lactoferrin, calprotectin and lipocalin-2, we found that they correlate well with each other [33].

**Table 1:** Demographic information for study participants (Children and caregivers; n = 375 at study start, except where otherwise noted).

***Child***	
*Gender (n*, *% male)*	180/375 (48%)
Age Months (mean ± SD)	14.3 ± 5.4 (n = 375)
Birth WAZ (mean ± SD) (by caregiver’s report)	-1.07 ± 1.7 (n = 365)
Breastfeeding (n, %*Yes*)	210/375 (56%)
Diarrhea on Day of Visit (n, %Yes)	10/375 (2.7%)
Caregiver (n = 375)*	
Age Years (mean ± SD)	26.3 ± 6.5 n = 373
Years Education	7.9 ± 3.0 n = 373
Age of 1^st^ Pregnancy	18.6 ± 4.2 n = 373

*Mother, n = 337;Grandmother, n = 27; Father, n = 9; other, n = 2.

**Table 2:** Frequencies of biomarker testing, including 13 tests on plasma, 4 on fecal and L/M absorption testing on urine as shown. *Of 326 children with samples obtained within 1 month of study start.

**Plasma Biomarkers**	# with Valid results*
SAA	281
LBP	281
sCD14	277
I-FABP	281
kyn	283
try	283
AdjFlicIgA	292
AdjLPSIgG	292
AdjFlicIgG	292
AdjLPSIgA	292
citrulline	283
LPS_Nutri_Enz (LUM)	289
Zonulin	288
** Fecal and Urine (L/M) Biomarkers**	
MPO	321
REG1	315
A1AT	289
Neo	234
L/M (and %L and %M)	274

In addition, followup anthropometry at 2–6 months after initial sampling enabled us to assess these biomarkers as predictors of subsequent growth. We also assessed their associations with each other as well as with separately studied comparisons of fecal lactoferrin, calprotectin and lipocalin, and plasma hsCRP, and claudin-15. Repeated Measures MANOVA analyses were used to model mean growth from Study Start to follow-up anthropometric measurement obtained within 2 to 6 months later by children with low and high levels of each biomarker while controlling for child age and gender. Interaction effects of gender with biomarker were also assessed for each model. Similarly interaction effects of study start growth status (stunting present or not) of biomarkers on growth were assessed, although these results were not significant.

Missing data analysis (Pearson correlations between known characteristics of participants retained or failed to return for followup or were missing biomarker results because of sample limitations) showed that children with caregivers having fewer years of education were missing more of three biomarkers (A1AT, Reg1, MPO, *r* ≤ -0.18) compared to their more educated counterparts. Boys were disproportionately missing A1AT results (r = 0.15 p = 0.003), older children were more likely to be missing Neopterin (r = 0.12 p = 0.017) while nonbreastfeeding (r = -0.10, p = 0.37) and more wasted children (r = -0.12, p = 0.026) are missing more L/Ms. To account for bias in followup, multiple regression was used to impute missing data for all biomarkers with at least 274 (73% of 375) complete data. Principle Components Analyses (PCA) were then used to combine related combinations of 11 barrier function, 2 intestinal inflammation and then 7 systemic biomarkers. These related sets of biomarkers were then correlated among each other as well as to children’s growth status and growth trajectory initially using Partial Pearson correlations and ultimately using Multiple Regression. All analyses predicting anthropometric status and growth trajectories included controlling for child age and gender.

## Results

### Markers of malnutrition (individual biomarkers at enrollment study start, ss)

**Fig 1:**
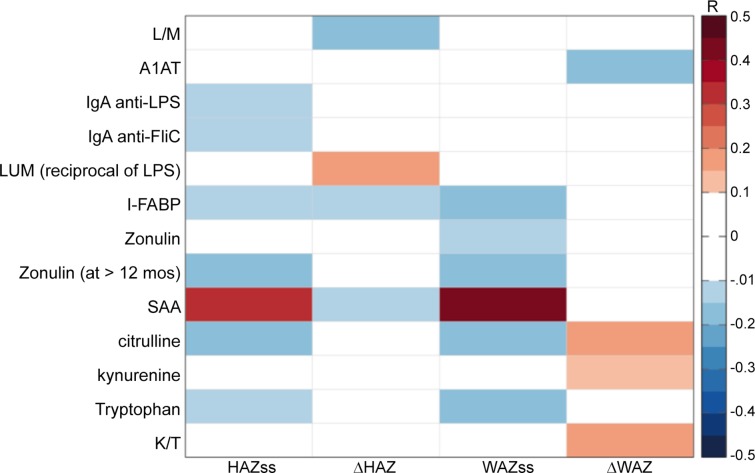
Heat map showing all significant partial Pearson correlations of barrier and systemic biomarkers with HAZ or WAZ at enrollment (ie. study start, ss) or with changes in (Δ) HAZ or WAZ. Significant correlations (at p<0.05; * = p<0.01) between biomarkers and growth, controlling for child age and gender. HAZ = height for age Z score; WAZ = weight for age z score; ss = study start; Δ = change in HAZ or WAZ at 2-6m followup; numbers range from 230 to 292 except for zonulin at age >12m where n = 172. Full r, p and df values are provided in S1 Table.

Several plasma biomarkers suggesting prior intestinal barrier disruption were significantly correlated with stunting or wasting, as assessed by HAZ or WAZ at study start, respectively, in children when they were first enrolled (ie at study start, ss). As shown in Fig 1 (and in S1 Table), these included plasma IgA anti-LPS antibody, zonulin (in all children for WAZ and, for HAZ in children >12 months old), and I-FABP. IgA anti-flagellin and anti-LPS antibodies (n = 292; *r* = 0.15, *p* = 0.011 and *r* = 0.14, *p* = 0.017) as well as intestinal fatty acid binding protein (I-FABP; n = 281; r = 0.14; *p* = 0.024) associate with worse stunting (i.e. lower HAZ SS scores) at the time of enrollment, suggesting greater prior exposure to LPS and release of an intestinal cytosolic protein, thus enterocyte damage, respectively. In addition, citrulline (as a marker of intestinal injury repair) and lower SAA (suggesting impaired defenses) correlated with stunting at enrollment.

### Predictors of subsequent growth (individual biomarkers with delta HAZ)

**Fig 2:**
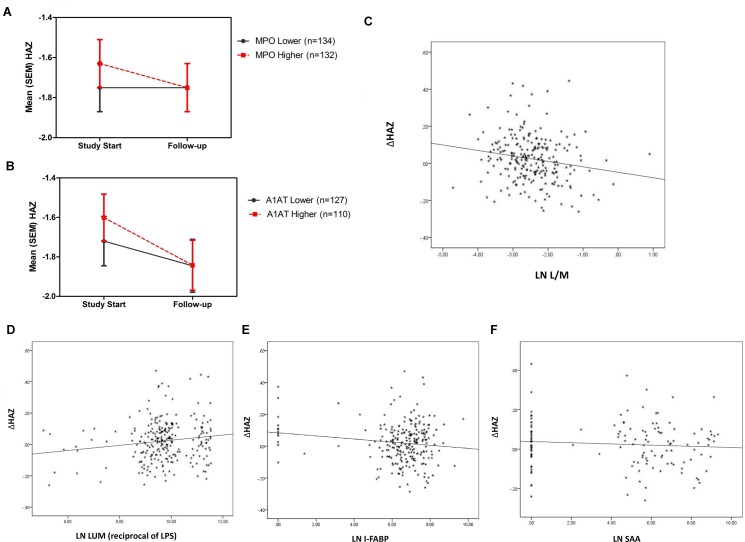
Fecal MPO, Fecal alpha-1-antitrypsin (A1AT), and plasma LPS, FABP and SAA each predicts subsequent growth impairment. a: For MPO, *p* = 0.028; n = 266 when correcting for age and gender, and independent of breastfeeding status (that showed no correlation in these 6-26m old children) and of age. b: For A1AT, n = 237; *p* = 0.042; and A1AT also correlates with “catchup WAZ” as well, *p* = 0.035 after correcting for age and gender. c: For urine L/M, higher values correlated (controlling for age and gender) with impaired growth (delta HAZ) (*r* = -0.173; *p* = 0.009; n = 230). d: For plasma LPS (ie lower LUM), higher values correlated with impaired growth (delta HAZ) (*r* = 0.151; *p* = 0.017; n = 251). e: For plasma FABP, higher values correlated with impaired growth (delta HAZ) (r = -0.134; *p* = 0.042; n = 231). f: For plasma SAA, higher values correlated with impaired growth (delta HAZ) (r = -0.132; p = 0.046; n = 231).

Several biomarkers of intestinal inflammation and barrier disruption (or “environmental enteropathy”) at the time of sampling were predictive of subsequent growth. Of importance, subsequent growth was significantly worse in those with higher fecal MPO or A1AT (Fig 2A and 2B), and growth impairment correlated with higher L/M, LPS, I-FABP and SAA (Fig 1 and Fig 2C–2F) in showing fecal and systemic markers of intestinal inflammation and barrier disruption or enteropathy).

Claudin-15 (Cdn-15), even with relatively few (n = 35–42) matching samples, significantly correlates with lower L/M, A1AT, Reg-1 and lipocalin-2, all markers (when lower) of 'better' mucosal integrity/barrier function, and all consistent with the previously recognized associations of Cdn-15 with healthy absorptive surface function and barrier function in animal models [34] (see S2 Table).

### Citrulliine predicts growth in girls and tryptophan in boys

**Fig 3:**
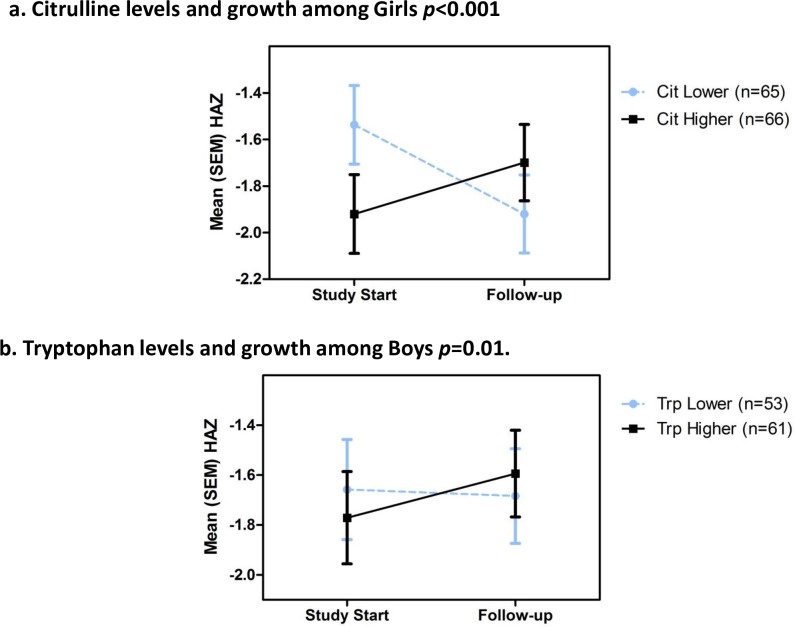
Plasma citrulline (in girls) and tryptophan (in boys) predicts subsequent better growth. A. citrulline (for which gender differs significantly) in girls (*p*<0.001; n = 131; median citrulline = 23.97 umol/L) and B. tryptophan in boys (*p* = 0.010; n = 114; median tryptophan = 66umol/L) predicts subsequent *better* growth.

Shown in Fig 3 (Fig 3A and 3B) are the associations of plasma citrulline (esp. in girls) and tryptophan (in boys) with improved growth. The linear association of plasma citrulline levels with weight gain is significant for all 246 children (*r* = 0.15, *p* = 0.018). Bivariate analyses show this is predominantly accounted for by improved growth among the 131 girls (*p*<0.001). Conversely, the association of plasma tryptophan with better subsequent growth was only seen in the 114 boys (*p* = 0.010). Furthermore, higher kynurenine/tryptophan ratios correlate with fecal MPO and systemic hsCRP (and negatively with citrulline) (S1 Table).

### Interaction of MPO and neopterin

**Fig 4:**
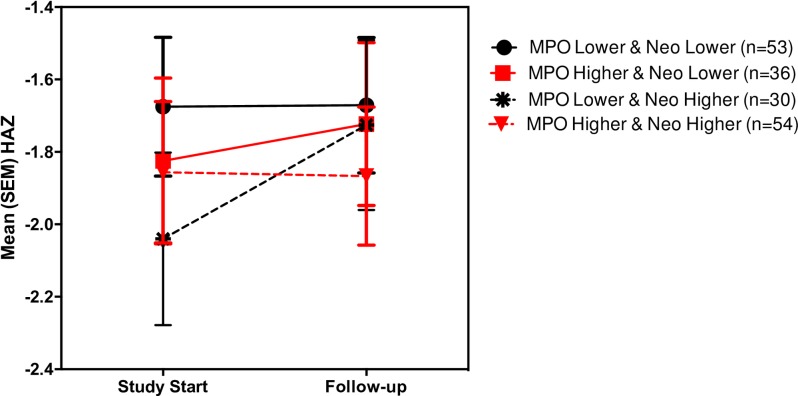
Repeated measures MANOVAs show interactions in predicting growth between MPO and Neo: high MPO when combined with high neopterin associate with poorest growth.

As shown in Fig 4 (Fig 4), Repeated Measures MANOVAs also shows an interaction effect in predicting growth: high MPO when combined with high neopterin associates with poorest growth. Thus, MPO combines with neopterin to show subsequent growth impairment; however, neopterin may also, in the absence of intestinal inflammation (ie low MPO), reflect “healthier” host cellular immune responses and thus correlate with better growth (again showing a significant *interaction* of MPO with neopterin).

### Clustering of biomarkers with each other

**Fig 5:**
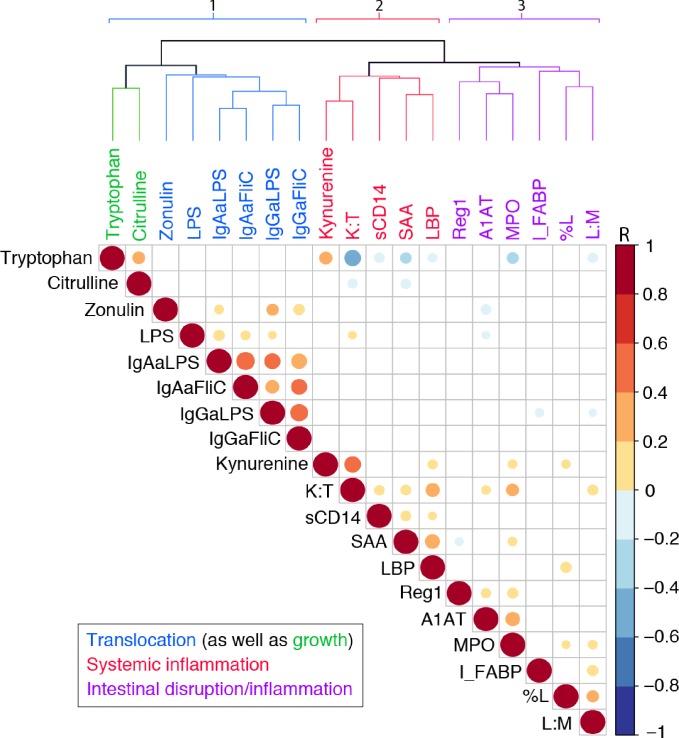
Cluster dendrogram with Pearson correlations among those biomarkers with ≥274 values showing three main groups: 1) translocation markers, LPS and IgA and IgG anti-LPS or FliC as well as zonulin and the 2 potential predictors of subsequent growth, tryptophan and citrulline; 2) predominantly systemic responses to disrupted barrier function and translocation; and 3) markers of specific intestinal barrier disruption or local intestinal inflammation. As discussed, groups 1 and 3 may predispose to group 2 systemic responses in associating with each other as shown in the heat map as would fit our concept of the pathogenesis of enteropathy.

The biomarkers tested tend to cluster as markers of intestinal barrier function (including LPS translocation), intestinal inflammation, or systemic inflammation as shown in Fig 5 (Fig 5) and in S2 Table. Specifically, in Fig 5 a cluster dendrogram with Pearson correlations among those biomarkers with ≥274 values are three main groups: the first representing the translocation markers, LPS and IgA and IgG anti-LPS or FliC as well as zonulin and the 2 predictors of subsequent growth, tryptophan and citrulline. The second group shows predominantly systemic responses to disrupted barrier function and translocation, while the third group are the markers of specific intestinal barrier disruption or local intestinal inflammation. Thus groups 1 and 3 likely lead to the group 2 systemic responses in associating with each other as well as fitting our concept of the pathogenesis of enteropathy. Shown in Fig 5 for the ≥274 biomarkers available within 1 month of enrollment and, more extensively for all tests done in S2 Table, fecal MPO correlates with fecal A1AT, and Reg-1, as well as with urinary L/M ratios, % lactulose and even with kynurenine, K/T ratios and (negatively) with tryptophan. Fecal MPO also correlated significantly with neopterin, albeit in smaller numbers available for neopterin at study start (n = 233; but with n = 279, r = 0.238, p<0.001 for all samples tested shown in S2 Table). Published separately using a subset of 77 of these fecal samples for comparative simultaneous studies of fecal MPO, lactoferrin (LF), calprotectin (FC) and Lipocalin-2 (Lcn-2) [33], we found that the neutrophil markers, MPO, LF and FC correlated best, but also, albeit less tightly, with Lcn-2. The full sample shows that fecal Reg-1 also correlated with A1AT and the intestinal epithelial biomarker Lcn-2 as well as with the inflammation biomarkers, lactoferrin, MPO or neopterin (Fig 1). In addition, fecal MPO correlates with several plasma biomarkers suggesting systemic inflammatory responses, including hsCRP (again, in S2 Table, with smaller numbers, but still significant at p<0.005; r = 0.238; n = 142), as well as with SAA, kynurenine, K/T and negatively with tryptophan.

### Pathway model, using clusters of biomarkers to predict malnutrition and growth

**Fig 6:**
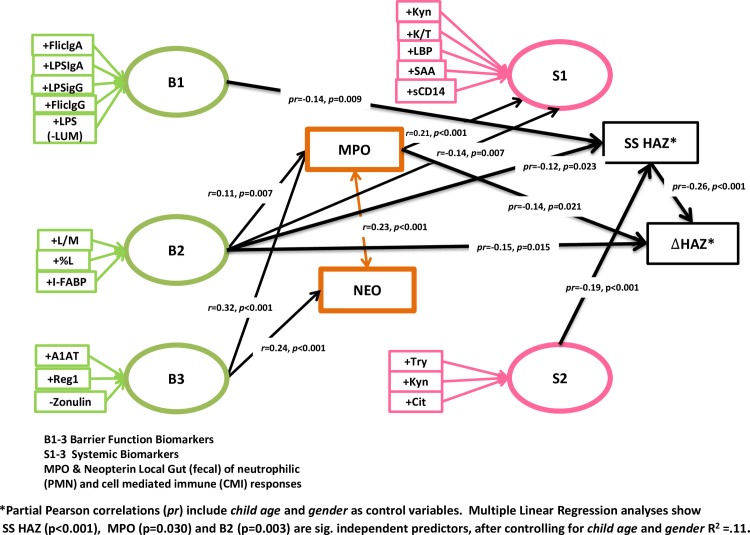
Path model, using Principle Components Analyses (Equamax rotation solution maximizing independence of groups) showing associations among 1) Barrier (green), 2) Local Gut (orange) and 3) Systemic (pink) sets of biomarkers, as well as their predictive utility regarding linear growth.

Shown in Fig 6 (Fig 6) are the results of principle components factor analyses to assess parsimonious linear pathways of grouped biomarkers as they relate to children’s growth status and subsequent growth. Available biomarker data with sufficient numbers for this principle component analysis that can be considered to reflect disruption of intestinal barrier function fall into 3 groups as shown, reflecting (B1) LPS ‘translocation’, (B2) absorptive function and epithelial cell damage (L/M and I-FABP), and (B3) further barrier disruption (ex Reg1 and A1AT) with tight junction modulation (zonulin). Most importantly, these factor analyses reveal associations of two pathways that associate with impaired subsequent growth: B2 (disrupted absorptive function and epithelial damage) and MPO (indicating intestinal inflammation); B2 additionally associates with MPO.

In addition, group B1 (markers of past and recent LPS translocation) associates with stunting at the time of enrollment (‘study start,’ or HAZss); and B2 barrier/absorptive disruption with the systemic cluster (S1: Kyn, K/T, LBP, sCD14, and SAA). Like B2 (barrier/absorptive disruption), the other cluster showing barrier disruption, B3, also associates separately with MPO, and with neopterin. Finally, stunting at enrollment highly significantly predicted ‘catch-up’ growth (ie stunted children indeed did show greater growth that has been termed ‘catch-up’ growth).

Multiple Linear Regression analysis showed HAZss (*p*<0.001), MPO (*p* = 0.041) and B2 (*p* = 0.021) are significant independent predictors of subsequent growth impairment (delta HAZ), after controlling for child age and gender (*R*^*2*^ = .11, p<0.001), or 11% of the growth impairment seen in these children.

## Discussion

The need for simple, sensitive biomarkers of environmental enteropathy (EE) in readily available fecal, blood or urine specimens lies in the magnitude of its impact and the need to understand and develop effective interventions. One in every three children in developing areas become stunted in their critically formative first 2 years of life, likely related at least in part to increased exposure to environmental pathogens, often as they are weaned in settings of inadequate water and sanitation [35–37]. The potential developmental consequences of enteropathy with or without overt diarrhea or stunting can be devastating to the full physical and neurocognitive development in one third of the world’s children growing up in impoverished areas. Thus early recognition and intervention in those at greatest risk is of paramount importance. Our findings focus attention on intestinal barrier and absorptive function and inflammation that might reflect increased functional gut permeability (assessed by L/M permeability testing and by serum zonulin levels) and on translocation of microbial products like LPS which then may trigger local and systemic inflammatory responses leading to further structural impairment of gut barrier (A1AT) and, ultimately, stunting growth that characterize EE.

With respect to biomarkers that associate with already stunted children, we find increased systemic IgA antibody against the microbial markers, LPS or FliC (suggesting prior translocation of microbial products), as well as Intestinal-FABP (a marker of enterocyte death) and, in children over 12 months of age, plasma zonulin all suggesting prior intestinal barrier disruption. Similarly, the negative association of the LPS ‘cluster’ (B1) with HAZss is seen in the Principle Components (Partial Pearson correlation) analyses shown in Fig 6. Conversely, the association of reduced SAA in stunted children may reflect an impairment in their host defense responses. In addition, greater citrulline and tryptophan levels in stunted children suggest their involvement in the needed intestinal injury repair or ‘catch-up’ growth, that is clearly greater in these stunted children.

In contrast, biomarkers that help ‘predict’ subsequent growth show the importance of an initial disruption of the intestinal barrier, translocation of LPS and local intestinal and inflammatory signaling as well as the importance of key building blocks like citrulline and tryptophan (albeit with opposite sex associations). Children with higher fecal MPO or fecal A1AT grow less well, and L/M, LPS (reciprocal of LUM), I-FABP and SAA all correlate significantly with impaired subsequent growth. Similarly, the interaction of the 2 fecal markers, MPO and neopterin shows additional power to predict subsequent growth impairment.

The differential association of select biomarkers in girls versus boys likely reflect differences in body composition and basal metabolic rates in males vs females, which are present well before the onset of puberty [38–46]. In addition, boys in low and middle income countries (LMICs) are at greater risk of stunting than girls—especially boys of families with the lowest socioeconomic resources (http://bmcpediatr.biomedcentral.com/articles/10.1186/1471-2431-7-17). Hence, just as children's growth is monitored using sex-adjusted anthropometric indices, the power of biomarkers to predict growth must be considered in the context of sex differences in growth. Further work to understand the biological basis of sexual dimorphisms in growth under adverse conditions is urgently needed.

Indeed, as shown in the cluster dendrogram in Fig 5 (Fig 5), the biomarkers themselves tend to cluster into 3 main groups, reflecting intestinal translocation, intestinal mucosal barrier disruption and inflammation, and systemic inflammatory responses that likely contribute to the potential long-term growth, developmental and metabolic consequences that have been attributed to repeated intestinal infections and “environmental enteropathy” in early childhood [5]. Those that indicate early tight junction effects (zonulin) cluster with the indicators of past or recent LPS translocation and even the potential “catch-up growth” markers, tryptophan and citrulline, while those that indicate intestinal cell and structural barrier disruption (I-FABP, L/M, %L, A1AT and Reg1) cluster with the marker of intestinal inflammation, MPO. Finally, markers of systemic acute phase or proinflammatory responses (SAA, Kynurenine, K/T, sCD14 and LBP) cluster to indicate the systemic responses to intestinal barrier disruption and inflammation that we postulate effect the troubling lasting growth, developmental and metabolic consequences noted above. Somewhat similar clustering of intestinal and systemic biomarkers has also been seen in children in Bangladesh [47].

Furthermore, our multivariate pathway analyses show that barrier function, intestinal inflammation and systemic markers are linearly associationed with each other as well as either previous stunting (ie HAZss at study start) or with subsequent growth (delta HAZ). As observed with single biomarker analyses, this more parsimonious pathway analysis elucidates the direct association of L/M, %L and I-FABP (B2, barrier/absorptive disruption) with growth impairment as well as its indirect association through MPO.

The antibody as well as ‘acute’ LPS cluster was associated with stunting at the time of sampling (as was seen with the IgA antibodies in the individual partial Pearson correlations shown in Table 2; and LPS, as reciprocal LUM, was correlated with subsequent growth impairment as well). As lower LUM values reflect high LPS-neutralizing enzyme activity and thus higher recent systemic LPS exposure, they correlate with subsequent growth impairment (Fig 2D), and with higher IgA or IgG anti-FliC or anti-LPS (*r* = 0.300, *p*<0.001 for IgA*α*FliC; *r* = 0.211, *p*<0.001 for IgA*α*LPS; *r* = 0.199, *p* = 0.001 for IgG*α*LPS; to *r* = 0.146; *p* = 0.014 for IgG*α*FliC). Thus, while IgA or IgG antibody against LPS or FliC likely reflect *prior* translocation of enteric bacterial components, hence stunting at enrollment; the more current LPS exposure, reflected by the reciprocal luminescence assay for LPS neutralizing activity correlates better with *subsequent* growth, even though it also correlates with anti-LPS and FliC antibody that had associated with pre-enrollment stunting. Therefore, this LPS-neutralizing enzyme activity assay may provide a better predictor of subsequent growth, irrespective of past stunting. Furthermore, it is clear that having antibody against LPS does *not protect* against subsequent stunting that is associated with recent evidence of LPS exposure. Several extensive studies have assessed LPS using plasma IgG anti-endotoxin core antibody (EndoCAb) as well as different measures of endotoxin and some, but not others have found impressive associations with impaired intestinal barrier function and growth [25, 28, 48]. These differences may relate to batch or other differences in available assays and hence are the reason we have used different assessments in this work. Taken together, however, consistent with our findings in this study, numerous studies suggest that different assessments of intestinal barrier disruption, and of local and systemic inflammation can be associated with impaired growth.

The third ‘barrier’ cluster (B3) of increased A1AT, Reg-1 and lower zonulin correlated with increased fecal MPO and with increased neopterin. In addition, the tendency for stunted children to experience “catch-up growth” was clearly shown in the association of stunting at study start (HAZss) with better subsequent growth as shown in Fig 6. The host attempt to “catch up” when challenged by growth impairing conditions has also been shown in intestinal cells, where “catch-up” intestinal cell kinase (ICK) is stimulated both *in vitro* and *in vivo* by even brief protein deprivation [49].

Intestinal inflammation can also further disrupt barrier function in a vicious cycle. Several fecal biomarkers have emerged as helpful tools in the assessment of intestinal inflammation or immune stimulation. As noted in our methods section, and having studied others (lactoferrin, calprotectin and lipocalin-2 with MPO) [33], we selected MPO and neopterin. The marker of intestinal epithelial cell repair, Reg-1B correlates with Lcn-2 [50], as it does with the marker of more severe barrier disruption, A1AT [51] in these fecal samples.

Although a poorly sensitive assay of structural intestinal barrier disruption, the serum protein that is inherently resistant to digestive degradation, A1AT has offered another biomarker that has shown correlations with epithelial destruction in previous studies [52]. Like lactulose absorption in the L/M test, these markers of barrier disruption could well represent key initiating or, with A1AT, later events in the development and progression of EE. A recent report by Brown et al [20] suggests that dietary deficiencies alone are insufficient to trigger inflammatory or villus architectural disruption without certain microbial components.

Plasma citrulline, an amino acid predominantly synthesized in proximal intestinal epithelial cells and only minimally present in the diet, appears to reflect healthy epithelium that is strikingly reduced in short bowel syndrome, AIDS enteropathy and other villus atrophy syndromes [53]. Its association with better subsequent growth, albeit with initial stunting suggests that it may play a key role in ‘catch-up’ growth, which, when possible is definitely greater in stunted children.

The acute phase protein, SAA (serum amyloid A protein) can be triggered by selected microbiota (segmented filamentous bacteria, SFB) [54] and has been shown to induce epigenetic alterations via JMJD3 that removes the methylation from the H3K27 repressor of the promotors of IL-23, G-CSF and TREM-1, and thus drives proinflammatory cytokine production [55, 56]. Hence, its association with fecal MPO, plasma hsCRP, sCD-14, LBP, kynurenine and K/T ratios (albeit with better HAZ at study start, perhaps reflecting more robust host responses, while also predicting poorer subsequent growth as shown in Fig 2F, and negatively with tryptophan suggest its potential role as not only a biomarker, but a potential pathway involved in the development of environmental enteropathy.

REG1B, the product of regenerating gene1 and marker of intestinal cell damage has been previously associated with growth failure in children in Bangladesh [50]. It has also been recently shown to cluster with sCD14, A1AT, MPO, calprotectin, and neopterin in these children linking environmental enteropathy (EE) with oral vaccine failure [57].

Soluble CD14 is secreted by monocytes and macrophages in response to LPS, and hence joins LBP in reflecting systemic responses to LPS translocation [58]. The marker of enterocyte death, intestinal fatty acid binding protein (I-FABP) [59] joins biomarkers of structural barrier disruption, including L/M and %L as well as A1AT and Reg1 as an indicator of monocyte responses to LPS bioactivity, sCD14 has also been associated with increased IL6, CRP, SAA, D-dimer and increased mortality in HIV-infected individuals [60], as well as directly measured LPS and I-FABP, all presumably reflecting an enteropathy with increased microbial (or microbial product) translocation [61]. The association of zonulin with LBP, IgG anti-LPS and anti-FliC, low tryptophan and malnutrition (lower SS WAZ for all children and, if greater than 12 months old, also SS HAZ) is consistent with the involvement of this tight junction modulator early in development of EE [62], while the reduced zonulin with increased A1AT or neopterin may reflect compensatory zonulin pathway down-regulation secondary to epithelial disruption. Interestingly in the Principle Components analyses (Fig 5), reduced zonulin with increased A1AT and Reg1 associated with both increased MPO and neopterin.

To our knowledge, this is the first evaluation of urinary claudin-15 as a biomarker for healthy gut function. Previous studies by Thuijls and colleagues used Western blotting to measure significant elevations of urinary claudin-3 in patients with active IBD versus those in remission or healthy controls [63]. Here, we successfully used ELISA to quantify urinary claudin-15 and uncovered inverse correlations with urinary L/M, and fecal A1AT, lipocalin-2, and Reg-1. Claudin-15 is highly expressed throughout the intestine [64] in an age-dependent manner [65]. Interestingly, lactating rats upregulate small intestinal Cld-15 expression in association with villous hypertrophy and enhanced calcium absorption [66], whereas Cld-15 ko display megaintestine and sodium deficiency and glucose malabsorption in the small intestine [67]. Taken together, this suggests that increased urinary Cld-15, like urinary mannitol, is a positive biomarker of gut health.

Potential pathways involved may thus include enteric pathogens (especially bacteria and protozoa) that compound dietary insufficiencies to disrupt intestinal mucus and epithelial barrier function, thereby enabling translocation of microbial LPS that triggers both local intestinal as well as systemic inflammatory signaling. Intestinal inflammation can also further disrupt barrier function in a vicious cycle. Both pathogen exposure and inadequate diet are products of poverty, with its concomitant inadequate water, sanitation and food. Our principle components analyses of biomarkers reveal that correlated groups of biomarkers that can be said to reflect barrier disruption have significant correlations with subsequent growth (as does initial HAZ) as well as with fecal and systemic biomarkers and selected metabolites.

Our findings in this report thus support the hypothesis that the systemic effects of potential intestinal barrier disruption and local inflammation on growth are likely occurring through effects of systemic inflammation that have indirect effects through impairing the hepatic synthesis of such key growth mediators as IGFBP-3 and IGF-1, as suggested by DeBoer et al [in press]. However, additional key pathways may also include effects via citrulline or via tryptophan metabolism. These key metabolites may become less available for intestinal injury repair because of increased demands or because of altered host or microbial metabolism or signaling. For example, the activation of indoleamine 2,3-dioxygenase-1 (IDO-1) by microbial translocation or LPS has been shown to be triggered in dendritic cells and monocytes to skew CD4+ T-cell differentiation away from T-helper (Th17) to regulatory T cells (Tregs) [68]. We thus find that lower plasma tryptophan levels not surprisingly associate with biomarkers of barrier disruption and intestinal and systemic inflammation, including higher L/M, zonulin, fecal MPO and lactoferrin, and higher hsCRP, SAA, sCD14, kynurenine and K/T ratios; while lower tryptophan is associated with lower citrulline. Indeed, in metabonomic studies of all metabolites in the urines of these children, we have recently found that tryptophan is likely depleted via both gut microbial metabolism (through the indole pathway to measureable increases in urinary 3-indole sulfate) as well as via the endogenous IDO/kynurenine pathway (as indicated by the increased nicotinic acid, N-methyl nicotinamide and 2-PY metabolites seen in the urine of these undernourished children, by WAZ or HAZ) [69]. In summary, we find that MPO, A1AT, LPS, L/M, I-FABP, and SAA, as well as MPO+ neopterin, and combinations of L/M and %L associate with impaired subsequent growth, with the reverse of these as well as citrulline (in girls) and tryptophan (in boys) offering potential predictors of better growth.

We conclude that fecal MPO and A1AT, and L/M, plasma LPS-neutralizing activity, I-FABP, SAA, hsCRP, citrulline and tryptophan provide promising biomarkers that associate with intestinal barrier disruption, local and systemic inflammation and potential catch-up growth involved in “environmental enteropathy.” These fecal, urinary and systemic biomarkers can prove useful in several ways. First, by improving our understanding of the pathogenesis and its long-term physical and cognitive consequences, they can elucidate its early recognition as well as innovative approaches to ameliorate its potentially devastating impact. Furthermore, they can provide targeted assessment tools with which to evaluate the effectiveness of novel interventions and improved approaches to controlling the potential lasting stunting and developmental consequences of frequent or repeated enteric infections in young children living in impoverished settings. Thus these key biomarkers can help complete the cycle of recognition, understanding, intervening and finally documenting causality of EE and its likely long-term consequences for healthy child development in areas of greatest need.

## Supporting Information

S1 File Supplementary Methods. (DOCX)

S1 Table Correlations between biomarkers and growth, controlling for child age and gender. (DOCX)

S2 Table Pearson correlations among all simultaneously sampled biomarkers (**p < 0.01 (yellow box) and *p < 0.05 (blue box)). Red font indicates *r*>0.25. Red font indicates *r*>0.25. Green, orange and pink shading represents barrier, gut and systemic biomarker groupings, respectively.
(DOCX)

## Abbreviations

EE: environmental enteropathy
WAZ: weight for age Z score
HAZ: height for age Z score
MPO: myeloperoxidase
hsCRP: high sensitivity C-reactive protein
SAA: serum amyloid A
LPS: lipopolysaccharide
I-FABP: intestinal fatty acid binding protein
Reg-1: regenerating gene 1β
sCD14: soluble cluster of differentiation 14
TJP1: tight junction protein gene encoding for ZO-1
IGFBP-3: insulin-like growth factor binding protein-3
IGF-1: insulin-like growth factor 1
A1AT: alpha-1-antitrypsin
Neo: neopterin
LF: lactoferrin
FC: fecal calprotectin
Calp: calprotectin (plasma)
Lcn-2: lipocalin-2, Cld-2 and Cld-15 = claudin-2 and -15
