# Supplementary material for: Biomarkers of Environmental Enteropathy, Inflammation, Stunting, and Impaired Growth in Children in Northeast Brazil
Source: PLoS One. 2016 Sep 30;11(9):e0158772. doi: 10.1371/journal.pone.0158772 (PMC5045163; doi:10.1371/journal.pone.0158772)
Supplement: S1 File — (DOCX) [file pone.0158772.s001.docx]

Supplementary materials:

**Anthropometric measurements**

Children were enrolled and followed to the extent possible at return visits over 2 to 5 months for anthropometric assessments during 12 months. Children were weighed using metric pediatric balances with a certified accuracy of 100g and length was measured using a market platform with a sliding footboard following standard protocol and quality control as previously described[1, 2].

**Assessment of Blood, Stool, and Urine Biomarkers**

Plasma, fecal and urine samples were collected and tested for potential biomarkers of intestinal or systemic inflammation or intestinal barrier dysfunction as listed in Table 1. Three hundred twenty one children provided fecal samples within 1 month of enrollment; 326 had urine specimens obtained as a part of the lactulose/mannitol permeability test and 292 provided initial blood plasma specimens for study. In addition, 252 who provided initial stool specimens returned to the clinic for follow-up after 2-5 months, thus enabling repeat anthropometric measurements to assess subsequent growth. All testing was performed according to manufacturer instructions.

Plasma specimens for SAA, I-FABP, LBP and sCD14 were diluted 50x, 100x, 1000x, 80x, 500x and 100x, respectively, following manufacturer recommendations. Plasma samples were separated and frozen at -80^o^C pending processing for biomarkers tested by Enzyme Linked ImmunoSorbent Assay (ELISA, using commercially available kits ) from Hycult Biotech (Uden, Netherlands) included high sensitivity C-reactive protein ( hsCRP ), serum amyloid A (SAA), LPS-binding protein (LBP), sCD14 and intestinal fatty acid-binding protein (I-FABP). In addition, kynurenine, tryptophan and citrulline were assayed by LC-MS/MS (Oregon Analytics, Eugene OR); LPS neutralizing activity was assayed by inhibition of LPS-induced luminescence (see below), and IgA and IgG antibodies against LPS or FliC were assayed per Ziegler et al. [3].

Serum zonulin levels were assessed in a semi-quantitative way by Western blot densitometry normalized by baseline value. Briefly, serum samples for each enrolled subject (70 μg per well) was run under nondentauring conditions on 4-20% Tris-Glycine gels (Invitrogen). Protein was transferred onto a PVDF membrane (Millipore) and probed with 1.5 μg/ml mouse monoclonal anti-human zonulin antibody (Bio-Rad). Bands were detected with Alexa Flor 680 conjugated goat anti-mouse IgG antibodies (ThermoFisher). Bands were visualized using the LI-COR system. Densitometry was measured using Image Studio software (LI-COR). All samples were normalized to a reference sample run separately on each gel.

Urinary claudin-15 assessment: Frozen urine specimens obtained for L/M measurements were thawed then aliquoted at spun at 3,000 rpm, 20 min, 4C, and the supernatant transferred to a new tube. Human Claudin-15 ELISA kits were purchased from Antibodies-Online (cat #ABIN1742862).  100 ul samples were assayed per the manufacturer’s protocol.

Stool specimens were collected from each child into a sterile container. All samples were aliquoted and stored at −20°C in cryovials until assay, when they were allowed to thaw at room and then specimens for alpha-1-antitrypsin, myeloperoxidase, neopterin and Reg1B were diluted 100x, 500x, 10x, 100x and 10000x, respectively in buffer with protease inhibitors (RIPA). Standard curves provided by the kit were utilized for analyte quantification.

The samples were centrifuged; subsequently the supernatants were used to measure the biomarkers. Fecal biomarkers tested included myeloperoxidase (MPO), product of regenerating gene 1β (Reg-1), alpha-1-antitrypsin (A1AT), and neopterin (Neo). MPO kits were from Immundiagnostik (Bensheim, Germany); A1AT kits were from Bioventor, Candler NC and used after dilution 1:500 in 0.9% saline; Neo kits were from Genway Biotech, San Diego CA; and Reg-1 kits were from TechLab, Blacksburg VA. The absorption was measured using an Epoch plate reader (Bio-tek Instruments, Inc.). All biomarker levels are expressed as μg/ml.

Urine was collected for lactulose:mannitol (L/M) absorption testing over 5h. The L/M test was administered to each infant at enrollment. The L/M test followed the MAL-ED protocol as previously described [4, 5]. Briefly, children were fasted for a minimum of 2 hours and water was permitted ad libitum after 30 minutes following administration of the carbohydrate solution (1g of mannitol plus 5g lactulose in 20mL). A dose of 2 mL/kg up to a maximum of 20 mL was administered for each child. Urine samples were collected for five hours and a total of urine volume with 1-2 drops of chlorhexidine (2.35%/ Sigma-Aldrich, St. Louis, MO) added as a preservative, and samples were aliquoted and stored at -80 °C until sugars were measured by high pressure liquid chromatograph with a pulsed amperometric detection (HPLC-PAD) system as previously described [5].

LPS Neutralizing Enzyme Assay:

Using quantitative bioassays, we analyzed the extent to which plasma samples neutralize lipopolysaccharides (LPS).  The LPS neutralizing enzymes in serum indicates an individual’s inflammatory status [6-8].  The presence of neutralizing activity in plasma provides a surrogate signal for current or very recent (e.g. within hours) circulating LPS and may reflect altered adaptive immune responses potentially linked to gut barrier dysfunction. To determine the LPS neutralizing activity of individual serum samples, we took advantage of the fact that bioactive LPS will induce the activation of the inflammatory transcription factor NF-κB in macrophages. Thus, activities that neutralize LPS activity should diminish the activation of NF-κB. We therefore mixed human plasma samples with a fixed concentration of *E. coli* LPS (10ng/mL), and this mixture was subsequently exposed to a murine macrophage cell line RAW264.7, which stably encodes an NF-κB dependent luciferase reporter gene. NF-κB activity was then assessed by the expression of luciferase. Specifically, RAW264.7 cells were grown in complete DMEM, containing 10% FBS, 2 mM L-glutamine, 1 mM sodium pyruvate, and combined antibiotics. Cells were plated at a density of 2 × 10^4^ cells/well in a half-volume 96-well plate and incubated overnight at 37°C under 5% CO2.  After incubation, 10μl of plasma sample was applied directly to each well, along with pure *E. coli* LPS.  Following a 4hr incubation, NF-κB activation as measured by luminescence, was performed using a SpectroMax plate reader at 470nm.   Luminescence data are normalized to standard curve values on each plate.  LPS-neutralizing enzyme activity was thus assessed as lower luminescence readings (LUM) representing NFκB activation by a standard LPS addition to the assay. Higher levels of LPS-neutralizing enzyme activity will result in less active LPS and therefore less NFκB activation. Therefore, this LUM value represents the *reciprocal* of the LPS ‘neutralizing’ or ‘detoxifying’ enzyme (presumably non-antibody, non TLR-4 mediated LPS neutralizing activity that increases within hours to days of increased LPS ‘exposure’,[9] hence the reciprocal of ‘acute’ LPS presence in the bloodstream).

**Statistical Methods**

Frequency tables and histogram plots with distribution characteristics were used to determine if natural log transformation of raw values was required before inclusion in parametric statistical analyses including Pearson correlations and Linear Regression equations. Repeated Measures MANOVA analyses were used to model mean growth from Study Start to follow-up anthropometric measurement obtained within 2.3 to 5 months later by children with low and high levels of each biomarker while controlling for child age and gender. Interaction effects of gender with biomarker were also assessed for each model. Similarly interaction effects of study start growth status (stunting present or not) of biomarkers on growth were assessed, although these results were not significant. A simple median cut ‘low’ or ‘high’ groups for each biomarker was calculated using study start values so that differences in growth related to biomarker could be assessed. Interaction effects were assessed among biomarkers with individually significant associations with growth. Additionally, Principle Components factor analyses were conducted using an Equimax rotation solution to group biomarkers in order to create a more parsimonious set of linear analyses. Biomarkers were included in this analysis using mean substitution when a minimum of 200 samples were available. SAS and SPSS software were used for analyses. Statistical significance was determined if *p* values were less than 0.05.

1. Lima AA, Oria RB, Soares AM, et al. Geography, population, demography, socioeconomic, anthropometry, and environmental status in the MAL-ED cohort and case-control study Sites in Fortaleza, Ceara, Brazil. Clin Infect Dis **2014**; 59 Suppl 4: S287-94.

2. Richard SA, Barrett LJ, Guerrant RL, Checkley W, Miller MA, Investigators M-EN. Disease surveillance methods used in the 8-site MAL-ED cohort study. Clin Infect Dis **2014**; 59 Suppl 4: S220-4.

3. Ziegler TR, Luo M, Estivariz CF, et al. Detectable serum flagellin and lipopolysaccharide and upregulated anti-flagellin and lipopolysaccharide immunoglobulins in human short bowel syndrome. American journal of physiology Regulatory, integrative and comparative physiology **2008**; 294(2): R402-10.

4. Guerrant RL, Lima AA, Barboza M, et al. Mechanisms and impact of enteric infections. Adv Exp Med Biol **1999**; 473: 103-12.

5. Kosek M, Guerrant RL, Kang G, et al. Assessment of environmental enteropathy in the MAL-ED cohort study: theoretical and analytic framework. Clin Infect Dis **2014**; 59 Suppl 4: S239-47.

6. Warren HS, Novitsky TJ, Ketchum PA, Roslansky PF, Kania S, Siber GR. Neutralization of bacterial lipopolysaccharides by human plasma. J Clin Microbiol **1985**; 22(4): 590-5.

7. Riveau GR, Novitsky TJ, Roslansky PF, Dinarello CA, Warren HS. Role of interleukin-1 in augmenting serum neutralization of bacterial lipopolysaccharide. J Clin Microbiol **1987**; 25(5): 889-92.

8. Warren HS, Knights CV, Siber GR. Neutralization and lipoprotein binding of lipopolysaccharides in tolerant rabbit serum. J Infect Dis **1986**; 154(5): 784-91.

9. Tan Y, Kagan JC. A cross-disciplinary perspective on the innate immune responses to bacterial lipopolysaccharide. Molecular cell **2014**; 54(2): 212-23.
