# Supplementary material for: Biomarkers of Environmental Enteropathy, Inflammation, Stunting, and Impaired Growth in Children in Northeast Brazil
Source: PLoS One. 2016 Sep 30;11(9):e0158772. doi: 10.1371/journal.pone.0158772 (PMC5045163; doi:10.1371/journal.pone.0158772)
Supplement: S1 Table — (DOCX) [file pone.0158772.s002.docx]

S1 Table. Correlations between biomarkers and growth, controlling for child age and gender.

|  | | HAZ SS | Velocity ΔHAZ | WAZ SS | Velocity ΔWAZ |
| --- | --- | --- | --- | --- | --- |
| LN_L_M_ratio_study_start_window | r | .004 | -.173 | .028 | -.053 |
|  | p | .949 | .009 | .645 | .418 |
|  | df | 270 | 226 | 270 | 229 |
| ULMPERLACT.1 | r | -.079 | -.054 | -.119 | -.093 |
|  | p | .191 | .421 | .050 | .157 |
|  | df | 270 | 226 | 270 | 229 |
| LN_ALA_studystart_window | r | -.050 | .005 | -.025 | -.163 |
|  | p | .401 | .940 | .670 | .012 |
|  | df | 285 | 233 | 285 | 235 |
| REG1_x10ngmL_study_start | r | .017 | .012 | -.030 | -.068 |
|  | p | .759 | .842 | .598 | .270 |
|  | df | 310 | 259 | 310 | 261 |
| adjLPSIgA_LN | r | -.140 | .007 | -.057 | -.017 |
|  | p | .017 | .913 | .333 | .785 |
|  | df | 288 | 249 | 288 | 251 |
| AdjLPSIgG | R | -.096 | .081 | -.065 | .018 |
|  | P | .102 | .202 | .273 | .771 |
|  | df | 287 | 249 | 287 | 251 |
| AdjFlicIgG | R | -.082 | .081 | -.032 | -.011 |
|  | P | .162 | .201 | .586 | .867 |
|  | df | 288 | 249 | 288 | 251 |
| AdjFlicIgA | R | -.150 | -.058 | -.081 | .045 |
|  | P | .011 | .364 | .170 | .480 |
|  | df | 288 | 249 | 288 | 251 |
| LPS_Nutri_Enz_LN_withinWindow | R | -.016 | .151 | .016 | .005 |
|  | P | .788 | .017 | .784 | .941 |
|  | df | 285 | 247 | 285 | 249 |
| Zonulin_final_LN | r | -.009 | -.006 | -.127 | .087 |
|  | p | .131 | .928 | .032 | .169 |
|  | df | 284 | 245 | 284 | 247 |
| **GE 12 mos**  Zonulin | r | -.170 | -.083 | -.179 | .132 |
|  | p | .027 | .319 | .020 | .112 |
|  | df | 168 | 143 | 168 | 143 |
| LN_MPO_Study_start_within_window | R | .001 | -.106 | .014 | -.018 |
|  | P | .984 | .086 | .804 | .770 |
|  | df | 316 | 262 | 316 | 264 |
| LN_Neo_studystart_window | R | .045 | .077 | .094 | -.017 |
|  | P | .492 | .291 | .155 | .814 |
|  | df | 229 | 189 | 229 | 191 |
| hs_CRP_LN_study_start | R | .012 | .003 | .049 | -.001 |
|  | P | .887 | .974 | .559 | .990 |
|  | df | 143 | 114 | 143 | 115 |
| LN_SAA | R | .346 | -.132 | .415 | -.058 |
|  | P | .000 | .046 | .000 | .382 |
|  | df | 277 | 227 | 277 | 230 |
| LN_CD14_study_start | R | .043 | .029 | .087 | .048 |
|  | P | .477 | .668 | .148 | .472 |
|  | df | 273 | 223 | 273 | 226 |
| LN_FABP | R | -.135 | -.134 | -.153 | .035 |
|  | P | .024 | .042 | .010 | .599 |
|  | df | 277 | 227 | 277 | 230 |
| LN_LBP | R | -.072 | .011 | -.068 | .098 |
|  | P | .230 | .869 | .260 | .139 |
|  | df | 277 | 226 | 277 | 229 |
| citrulline_umolpL_marg_jan2015 | R | -.155 | .072 | -.161 | .151 |
|  | P | .009 | .268 | .007 | .018 |
|  | df | 279 | 240 | 279 | 242 |
| kyn_umolpL | R | -.100 | .009 | -.080 | .133 |
|  | P | .095 | .884 | .181 | .039 |
|  | df | 279 | 240 | 279 | 242 |
| try_umolpL | R | -.120 | .088 | -.151 | -.023 |
|  | P | .045 | .172 | .011 | .717 |
|  | df | 279 | 240 | 279 | 242 |
| LN_KT | R | .011 | -.070 | .058 | .157 |
|  | P | .855 | .281 | .333 | .014 |
|  | df | 279 | 240 | 279 | 242 |
| hs_CALPRO_result_study_start_plasma | R | .011 | .049 | .049 | -.129 |
|  | P | .906 | .631 | .589 | .202 |
|  | df | 124 | 95 | 124 | 97 |
