# Supplementary material for: Biomarkers of Environmental Enteropathy, Inflammation, Stunting, and Impaired Growth in Children in Northeast Brazil
Source: PLoS One. 2016 Sep 30;11(9):e0158772. doi: 10.1371/journal.pone.0158772 (PMC5045163; doi:10.1371/journal.pone.0158772)
Supplement: S2 Table — Red font indicates r>0.25. Red font indicates r>0.25. Green, orange and pink shading represents barrier, gut and systemic biomarker groupings, respectively. (DOCX) [file pone.0158772.s003.docx]

| **1. L/M n=274** | r | 1 |  |  |  |  |  |  |  |  |  |  |  |  |  |  |  |  |  |  |  |  |  |  | |  |  |
| --- | --- | --- | --- | --- | --- | --- | --- | --- | --- | --- | --- | --- | --- | --- | --- | --- | --- | --- | --- | --- | --- | --- | --- | --- | --- | --- | --- |
|  | **p** |  |  |  |  |  |  |  |  |  |  |  |  |  |  |  |  |  |  |  |  |  |  |  | |  |  |
|  | **n** | **274** |  |  |  |  |  |  |  |  |  |  |  |  |  |  |  |  |  |  |  |  |  |  | |  |  |
| **2. % L n=274** | **r** | **0.259**** | **1** |  |  |  |  |  |  |  |  |  |  |  |  |  |  |  |  |  |  |  |  |  | |  |  |
|  | **p** | **0.000** |  |  |  |  |  |  |  |  |  |  |  |  |  |  |  |  |  |  |  |  |  |  | |  |  |
|  | **n** | **274** | **274** |  |  |  |  |  |  |  |  |  |  |  |  |  |  |  |  |  |  |  |  |  | |  |  |
| **3. A1AT n=338** | **r** | **0.086** | **0.083** | **1** |  |  |  |  |  |  |  |  |  |  |  |  |  |  |  |  |  |  |  |  | |  |  |
|  | **p** | **0.172** | **0.189** |  |  |  |  |  |  |  |  |  |  |  |  |  |  |  |  |  |  |  |  |  | |  |  |
|  | **n** | **255** | **255** | **338** |  |  |  |  |  |  |  |  |  |  |  |  |  |  |  |  |  |  |  |  | |  |  |
| **4. LPC n=77** | **r** | **0.222** | **-0.039** | **.289*** | **1** |  |  |  |  |  |  |  |  |  |  |  |  |  |  |  |  |  |  |  | |  |  |
|  | **p** | **0.099** | **0.773** | **0.014** |  |  |  |  |  |  |  |  |  |  |  |  |  |  |  |  |  |  |  |  | |  |  |
|  | **n** | **56** | **56** | **72** | **77** |  |  |  |  |  |  |  |  |  |  |  |  |  |  |  |  |  |  |  | |  |  |
| **5. Reg1 n=315** | **r** | **-0.065** | **0.074** | **.212^**^** | **.273^*^** | **1** |  |  |  |  |  |  |  |  |  |  |  |  |  |  |  |  |  |  | |  |  |
|  | **p** | **0.322** | **0.264** | **0.000** | **0.023** |  |  |  |  |  |  |  |  |  |  |  |  |  |  |  |  |  |  |  | |  |  |
|  | **n** | **233** | **233** | **306** | **69** | **315** |  |  |  |  |  |  |  |  |  |  |  |  |  |  |  |  |  |  | |  |  |
| **6. LPSIgA n=292** | **r** | **0.001** | **-0.048** | **-0.027** | **0.217** | **0.072** | **1** |  |  |  |  |  |  |  |  |  |  |  |  |  |  |  |  |  | |  |  |
|  | **p** | **0.986** | **0.476** | **0.662** | **0.093** | **0.264** |  |  |  |  |  |  |  |  |  |  |  |  |  |  |  |  |  |  | |  |  |
|  | **n** | **226** | **226** | **265** | **61** | **244** | **292** |  |  |  |  |  |  |  |  |  |  |  |  |  |  |  |  |  | |  |  |
| **7. LPSigG n=291** | **r** | **-.151^*^** | **-0.046** | **-0.084** | **-0.02** | **0.001** | **.463^**^** | **1** |  |  |  |  |  |  |  |  |  |  |  |  |  |  |  |  | |  |  |
|  | **p** | **0.023** | **0.489** | **0.176** | **0.881** | **0.989** | **0.000** |  |  |  |  |  |  |  |  |  |  |  |  |  |  |  |  |  | |  |  |
|  | **n** | **225** | **225** | **264** | **61** | **243** | **291** | **291** |  |  |  |  |  |  |  |  |  |  |  |  |  |  |  |  | |  |  |
| **8. FliclgG n=292** | **r** | **-0.114** | **-0.098** | **-.125^*^** | **0.157** | **-0.061** | **.461^**^** | **.604^**^** | **1** |  |  |  |  |  |  |  |  |  |  |  |  |  |  |  | |  |  |
|  | **p** | **0.086** | **0.142** | **0.042** | **0.227** | **0.34** | **0.000** | **0.000** |  |  |  |  |  |  |  |  |  |  |  |  |  |  |  |  | |  |  |
|  | **n** | **226** | **226** | **265** | **61** | **244** | **292** | **291** | **292** |  |  |  |  |  |  |  |  |  |  |  |  |  |  |  | |  |  |
| **9. FliclgA n=292** | **r** | **0.053** | **0.052** | **-0.081** | **0.142** | **-0.027** | **.699^**^** | **.388^**^** | **.509^**^** | **1** |  |  |  |  |  |  |  |  |  |  |  |  |  |  | |  |  |
|  | **p** | **0.432** | **0.437** | **0.186** | **0.275** | **0.673** | **0.000** | **0.000** | **0.000** |  |  |  |  |  |  |  |  |  |  |  |  |  |  |  | |  |  |
|  | **n** | **226** | **226** | **265** | **61** | **244** | **292** | **291** | **292** | **292** |  |  |  |  |  |  |  |  |  |  |  |  |  |  | |  |  |
| **10. LUM n=289** | **r** | **0.01** | **0.059** | **0.1** | **-0.012** | **-0.014** | **-.211^**^** | **-.199^**^** | **-.146^*^** | **-.300**** | **1** |  |  |  |  |  |  |  |  |  |  |  |  |  | |  |  |
|  | **p** | **0.885** | **0.376** | **0.106** | **0.927** | **0.824** | **0.000** | **0.001** | **0.014** | **0.000** |  |  |  |  |  |  |  |  |  |  |  |  |  |  | |  |  |
|  | **n** | **224** | **224** | **262** | **62** | **243** | **283** | **282** | **283** | **283** | **289** |  |  |  |  |  |  |  |  |  |  |  |  |  | |  |  |
| **11. Zonulin n=288** | **r** | **-0.049** | **-0.011** | **-.142**^*^ | **-0.237** | **-0.065** | **0.080** | **.294**^**^ | **.275**^**^ | **.135**^*^ | **-0.059** | **1** |  |  |  |  |  |  |  |  |  |  |  |  | |  |  |
|  | **p** | **0.466** | **0.868** | **0.022** | **0.062** | **0.317** | **0.183** | **0.000** | **0.000** | **0.023** | **0.323** |  |  |  |  |  |  |  |  |  |  |  |  |  | |  |  |
|  | **n** | **220** | **220** | **261** | **63** | **241** | **282** | **281** | **282** | **282** | **285** | **288** |  |  |  |  |  |  |  |  |  |  |  |  | |  |  |
| **12. CD15 n=46** | **r** | **-.342**^*^ | **-0.105** | **-.427**^**^ | **-.827**^*^ | **-.362**^*^ | **0.304** | **0.086** | **0.075** | **0.113** | **-0.309** | **0.189** | **1** |  |  |  |  |  |  |  |  |  |  |  | |  |  |
|  | **p** | **0.033** | **0.524-** | **0.005** | **0.011** | **0.019** | **0.076** | **0.624** | **0.668** | **0.519** | **0.071** | **0.276** |  |  |  |  |  |  |  |  |  |  |  |  | |  |  |
|  | **n** | **39** | **39** | **42** | **8** | **42** | **35** | **35** | **35** | **35** | **35** | **35** | **46** |  |  |  |  |  |  |  |  |  |  |  | |  |  |
| **13. MPO n=351** | **r** | **.183^**^** | **.123^*^** | **.304^**^** | **.488^**^** | **.245^**^** | **-0.045** | **-0.051** | **-0.015** | **-0.039** | **0.007** | **0.003** | **-0.020** | **1** |  |  |  |  |  |  |  |  |  |  | |  |  |
|  | **p** | **0.003** | **0.048** | **0.000** | **0.000** | **0.000** | **0.455** | **0.403** | **0.806** | **0.524** | **0.905** | **0.966** | **0.900** |  |  |  |  |  |  |  |  |  |  |  | |  |  |
|  | **n** | **261** | **261** | **337** | **77** | **315** | **275** | **274** | **275** | **275** | **272** | **271** | **43** | **351** |  |  |  |  |  |  |  |  |  |  | |  |  |
| **14. LF n=77** | **r** | **.297^*^** | **-0.015** | **.438^**^** | **.460^**^** | **.247^*^** | **0.01** | **-0.076** | **0.248** | **0.04** | **-0.029** | **-0.174** | **-0.349** | **.568^**^** | **1** |  |  |  |  |  |  |  |  |  | |  |  |
|  | **p** | **0.026** | **0.91** | **0.000** | **0.000** | **0.040** | **0.942** | **0.562** | **0.054** | **0.76** | **0.823** | **0.174** | **0.397** | **0.000** |  |  |  |  |  |  |  |  |  |  | |  |  |
|  | **n** | **56** | **56** | **72** | **77** | **69** | **61** | **61** | **61** | **61** | **62** | **63** | **8** | **77** | **77** |  |  |  |  |  |  |  |  |  | |  |  |
| **15.CPT_fecal n=77** | **r** | **0.174** | **0.107** | **.256^*^** | **.483^**^** | **0.062** | **-0.093** | **0.016** | **0.191** | **0.095** | **-0.102** | **-0.005** | **-0.427** | **.654^**^** | **.614^**^** | **1** |  |  |  |  |  |  |  |  | |  |  |
|  | **p** | **0.199** | **0.433** | **0.030** | **0.000** | **0.612** | **0.476** | **0.905** | **0.14** | **0.465** | **0.430** | **0.970** | **0.292** | **0.000** | **0.000** |  |  |  |  |  |  |  |  |  | |  |  |
|  | **n** | **56** | **56** | **72** | **77** | **69** | **61** | **61** | **61** | **61** | **62** | **63** | **8** | **77** | **77** | 77 |  |  |  |  |  |  |  |  | |  |  |
| **16. Neo n=280** | **r** | **0.037** | **-0.026** | **.208^**^** | **0.19** | **.211^**^** | **-0.034** | **-0.053** | **-0.054** | **-0.014** | **-0.021** | **-.147**^*^ | **-0.006** | **.238^**^** | **0.163** | 0.166 | **1** |  |  |  |  |  |  |  | |  |  |
|  | **p** | **0.593** | **0.706** | **0.001** | **0.147** | **0.001** | **0.617** | **0.44** | **0.425** | **0.839** | **0.761** | **0.032** | **0.972** | **0.000** | **0.212** | 0.204 |  |  |  |  |  |  |  |  | |  |  |
|  | **n** | **207** | **207** | **271** | **60** | **255** | **218** | **217** | **218** | **218** | **217** | **215** | **34** | **279** | **60** | 60 | **280** |  |  |  |  |  |  |  | |  |  |
| **17. hsCRP n=151** | **r** | **-0.037** | **.197^*^** | **-0.07** | **.338^*^** | **-0.167** | **0.078** | **0.025** | **0.081** | **0.079** | **-0.068** | **0.076** | **-0.261** | **.238^**^** | **.406^*^** | .455^**^ | **0.094** | **1** |  |  |  |  |  |  | |  |  |
|  | **p** | **0.698** | **0.037** | **0.414** | **0.044** | **0.055** | **0.385** | **0.779** | **0.368** | **0.383** | **0.447** | **0.396** | **0.296** | **0.004** | **0.014** | **0.005** | **0.308** |  |  |  |  |  |  |  | |  |  |
|  | **n** | **112** | **112** | **139** | **36** | **133** | **125** | **124** | **125** | **125** | **127** | **127** | **18** | **142** | **36** | **36** | **119** | **151** |  |  |  |  |  |  | |  |  |
| **18. SAA n=281** | **r** | **0.095** | **0.001** | **-0.07** | **0.115** | **-0.11** | **0.019** | **0.030** | **0.063** | **0.027** | **-0.044** | **0.111** | **-0.288** | **.144^*^** | **0.021** | **0.066** | **0.108** | **.560^**^** | **1** |  |  |  |  |  | |  |  |
|  | **p** | **0.172** | **0.987** | **0.264** | **0.393** | **0.090** | **0.769** | **0.650** | **0.335** | **0.684** | **0.502** | **0.086** | **0.089** | **0.019** | **0.880** | **0.626** | **0.112** | **0** |  |  |  |  |  |  | |  |  |
|  | **n** | **210** | **210** | **256** | **57** | **239** | **236** | **235** | **236** | **236** | **239** | **239** | **36** | **264** | **57** | **57** | **217** | **146** | **281** |  |  |  |  |  | |  |  |
| **19. sCD14 n=277** | **r** | **0.023** | **-0.052** | **0.015** | **0.130** | **-0.101** | **-0.073** | **-0.011** | **0.047** | **0.034** | **0.008** | **0.085** | **-0.027** | **0.084** | **0.095** | **0.178** | **-0.076** | **.232^**^** | **.185^**^** | **1** |  |  |  |  | |  |  |
|  | **p** | **0.741** | **0.461** | **0.813** | **0.338** | **0.123** | **0.265** | **0.867** | **0.473** | **0.61** | **0.900** | **0.194** | **0.872** | **0.176** | **0.486** | **0.188** | **0.268** | **0.005** | **0.002** |  |  |  |  |  | |  |  |
|  | **n** | **206** | **206** | **252** | **56** | **233** | **232** | **231** | **232** | **232** | **235** | **235** | **37** | **259** | **56** | **56** | **216** | **146** | **275** | **277** |  |  |  |  | |  |  |
| **20. I FABP n=281** | **r** | **0.251^**^** | **0.023** | **0.004** | **0.052** | **-0.035** | **0.024** | **-0.118** | **-0.05** | **0.064** | **-0.005** | **-0.002** | **-0.018** | **-0.074** | **0.12** | **-0.018** | **-0.130** | **-0.119** | **-0.059** | **-0.003** | **1** |  |  |  | |  |  |
|  | **p** | **0.000** | **0.739** | **0.953** | **0.703** | **0.59** | **0.712** | **0.071** | **0.441** | **0.327** | **0.939** | **0.970** | **0.918** | **0.233** | **0.374** | **0.895** | **0.057** | **0.153** | **0.320** | **0.958** |  |  |  |  | |  |  |
|  | **n** | **210** | **210** | **256** | **57** | **239** | **236** | **235** | **236** | **236** | **239** | **239** | **36** | **264** | **57** | **57** | **217** | **146** | **281** | **275** | **281** |  |  |  | |  |  |
| **21. LBP n=281** | **r** | **-0.043** | **.157^*^** | **-0.024** | **0.167** | **0.032** | **.135^*^** | **0.092** | **0.054** | **.143*** | **-0.092** | **.135**^*^ | **-0.243** | **0.097** | **0.085** | **0.091** | **0.007** | **.613^**^** | **.426^**^** | **.159^**^** | **0.033** | **1** |  |  | |  |  |
|  | **p** | **0.539** | **0.023** | **0.706** | **0.211** | **0.629** | **0.039** | **0.159** | **0.407** | **0.028** | **0.158** | **0.037** | **0.148** | **0.118** | **0.526** | **0.496** | **0.919** | **0** | **0** | **0.008** | **0.587** |  |  |  | |  |  |
|  | **n** | **209** | **209** | **256** | **58** | **237** | **235** | **234** | **235** | **235** | **238** | **238** | **37** | **263** | **58** | **58** | **217** | **146** | **279** | **275** | **279** | **281** |  |  | |  |  |
| **22. Cit n=283** | **r** | **-0.098** | **-0.012** | **-.143^*^** | **0.094** | **-0.098** | **-0.012** | **0.085** | **0.024** | **0.006** | **0.067** | **0.004** | **0.024** | **-0.059** | **0.131** | **0.149** | **-0.117** | **-0.162** | **-.170^**^** | **-0.068** | **-0.05** | **-.131^*^** | **1** |  | |  |  |
|  | **p** | **0.148** | **0.857** | **0.022** | **0.473** | **0.135** | **0.848** | **0.159** | **0.692** | **0.923** | **0.264** | **0.942** | **0.893** | **0.341** | **0.313** | **0.25** | **0.09** | **0.07** | **0.009** | **0.303** | **0.443** | **0.044** |  |  | |  |  |
|  | **n** | **218** | **218** | **256** | **61** | **236** | **278** | **277** | **278** | **278** | **281** | **283** | **35** | **266** | **61** | **61** | **211** | **126** | **237** | **233** | **237** | **236** | **283** |  | |  |  |
| **23. Try n=283** | **r** | **-.154^*^** | **.147^*^** | **-0.073** | **-0.110** | **0.001** | **-0.081** | **-0.026** | **-0.073** | **-0.04** | **0.076** | **-.135**^*^ | **0.178** | **-.154^*^** | **-.257^*^** | **-0.197** | **0.008** | **-.277^**^** | **-.192^**^** | **-.161^*^** | **0.009** | **-.169^**^** | **.311^**^** | **1** | |  |  |
|  | **p** | **0.023** | **0.03** | **0.248** | **0.399** | **0.984** | **0.179** | **0.672** | **0.226** | **0.508** | **0.203** | **0.023** | **0.305** | **0.012** | **0.046** | **0.129** | **0.911** | **0.002** | **0.003** | **0.014** | **0.886** | **0.009** | **0** |  | |  |  |
|  | **n** | **218** | **218** | **256** | **61** | **236** | **278** | **277** | **278** | **278** | **281** | **283** | **35** | **266** | **61** | **61** | **211** | **126** | **237** | **233** | **237** | **236** | **283** | **283** | |  |  |
| **24. Kyn n=283** | **r** | **.164^*^** | **0.053** | **0.061** | **0.187** | **0.008** | **0.108** | **-0.069** | **0.034** | **0.112** | **-0.027** | **0.047** | **-.348**^*^ | **.235^**^** | **0.214** | **.305^*^** | **0.061** | **.340^**^** | **.224^**^** | **.205^**^** | **0.065** | **.316^**^** | **-.160^**^** | **-.581^**^** | | **1** |  |
|  | **p** | **0.015** | **0.434** | **0.329** | **0.148** | **0.898** | **0.073** | **0.25** | **0.577** | **0.062** | **0.657** | **0.428** | **0.040** | **0.000** | **0.098** | **0.017** | **0.376** | **0.000** | **0.001** | **0.002** | **0.321** | **0** | **0.007** | **0** | |  |  |
|  | **n** | **218** | **218** | **256** | **61** | **236** | **278** | **277** | **278** | **278** | **281** | **283** | **35** | **266** | **61** | **61** | **211** | **126** | **237** | **233** | **237** | **236** | **283** | **283** | | **283** |  |
| **25. K/T n=283** | **r** | **.142^*^** | **0.042** | **0.055** | **0.151** | **0.023** | **.126^*^** | **-0.065** | **0.037** | **.133*** | **-0.040** | **0.058** | **-0.326** | **.202^**^** | **0.183** | **.268^*^** | **0.054** | **.313^**^** | **.220^**^** | **.183^**^** | **0.042** | **.304^**^** | **-.156^**^** | **-.538^**^** | | **.963^**^** | **1** |
|  | **p** | **0.037** | **0.536** | **0.384** | **0.245** | **0.726** | **0.036** | **0.280** | **0.54** | **0.026** | **0.505** | **0.331** | **0.056** | **0.001** | **0.158** | **0.037** | **0.432** | **0.000** | **0.001** | **0.005** | **0.518** | **0** | **0.008** | **0** | | **0** |  |
|  | **n** | **218** | **218** | **256** | **61** | **236** | **278** | **277** | **278** | **278** | **281** | **283** | **35** | **266** | **61** | **61** | **211** | **126** | **237** | **233** | **237** | **236** | **283** | **283** | | **283** | **283** |
|  |  | **1. L/M** | **2. % L** | **3. A1AT** | **4. LPC-2** | **5. Reg-1** | **6. LPSIgA** | **7. LPSigG** | **8. FliclgG** | **9. FliclgA** | **10. LUM** | **11. Zonulin** | **12. CD15** | **13. MPO** | **14. LF** | **15. CPT_fecal** | **16. Neo** | **17. hsCRP** | **18. SAA** | **19. CD14** | **20. FABP** | **21. LBP** | **22. Cit** | | **23. Try** | **24. Kyn** | **25. K/T** |

**Supplementary Table S2.** Pearson correlations among all simultaneously sampled biomarkers (***p* < 0.01 (yellow box) and **p* < 0.05 (blue box)). Red font indicates *r*>0.25. Red font indicates *r*>0.25. Green, orange and pink shading represents barrier, gut and systemic biomarker groupings , respectively.
